# Regulation by Ascorbic Acid and HOO• Radicals of Extracellular DNA Network Formation and Internalization Activity of Mononuclear Cells

**DOI:** 10.17691/stm2026.18.3.05

**Published:** 2026-06-30

**Authors:** K.M. Fiallos Barrionuevo, D. Sanogo, E.N. Gorshkova, I.P. Ivanova

**Affiliations:** 1 PhD Student, Department of Molecular Biology and Immunology; National Research Lobachevsky State University of Nizhny Novgorod, 23 Prospekt Gagarina, Nizhny Novgorod, 603022, Russia; 2 PhD Student, Department of Molecular Biology and Immunology; National Research Lobachevsky State University of Nizhny Novgorod, 23 Prospekt Gagarina, Nizhny Novgorod, 603022, Russia; 3 PhD, Associate Professor, Department of Molecular Biology and Immunology; National Research Lobachevsky State University of Nizhny Novgorod, 23 Prospekt Gagarina, Nizhny Novgorod, 603022, Russia; 4 DSc, Professor, Department of Molecular Biology and Immunology; National Research Lobachevsky State University of Nizhny Novgorod, 23 Prospekt Gagarina, Nizhny Novgorod, 603022, Russia

**Keywords:** HOO• radical, ascorbic acid, mononuclear cells, extracellular DNA networks, internalization, intracellular reactive oxygen species, fluorescence

## Abstract

**The aim of the study** was to identify the regulatory potential of ascorbic acid and HOO• radicals in the formation of extracellular DNA networks and the internalization activity of mononuclear cells from rat blood and spleen.

**Materials and Methods:**

The isolated mononuclear cells from rat blood and spleen were exposed to ascorbic acid (10 and 50 μM), hydroperoxyl radicals (HOO•), and their combination. The following parameters were assessed: membrane toxicity (using the trypan blue exclusion test), internalization activity with latex particles (endocytosis), extracellular DNA networks formation, intracellular reactive oxygen species production kinetics (flow cytometry with the intracellular fluorescent probe dihydrorhodamine 123, DHR123), tryptophan fluorescence, glycated protein levels, and cellular redox balance (NAD+ and NADH).

**Results:**

Ascorbic acid at the concentrations of 10 and 50 μM suppressed the formation of extracellular DNA networks. At a concentration of 50 μM, it exerted a membrane-toxic effect, reduced tryptophan fluorescence, increased glycated protein levels, and shifted the redox balance toward oxidation (increased NAD+ and decreased NADH). HOO• radicals at concentrations ≥200 μM demonstrated concentration-dependent effects: stimulation of endocytosis, increased formation of extracellular DNA networks, and elevated intracellular production of reactive oxygen species. At a concentration of 600 μM, HOO• radicals reduced tryptophan fluorescence and depleted both NAD+ and NADH levels. The stimulatory effects on endocytosis and extracellular DNA network formation in response to HOO• radicals were the most pronounced in splenic mononuclear cells.

The analysis of the combined action of HOO• radicals and ascorbic acid showed that ascorbic acid at a concentration of 10 μM protected cells from the membrane-toxic effects of HOO• radicals and reduced the formation of extracellular DNA networks induced by HOO• radicals. The ascorbic acid at a concentration of 50 μM enhanced the membrane-toxic effect of HOO• radicals and reduced the internalization activity of mononuclear cells.

**Conclusion:**

HOO• radicals at concentrations ≥200 μM stimulated the internalization activity of mononuclear cells and the intracellular generation of reactive oxygen species, whereas HOO• radicals at 600 μM caused a decrease in both oxidized and reduced coenzyme levels and increased the formation of extracellular DNA networks. Ascorbic acid at a concentration of 10 μM exhibited antioxidant properties and protected cells from the damaging effects of HOO• radicals, while at 50 μM it acted as a synergistic pro-oxidant, enhancing the membrane-toxic effect of the radicals and reducing the internalization activity of cells.

## Introduction

Ascorbic acid is a typical modulator of redox processes and a recognized antioxidant that directly scavenges reactive oxygen species (ROS), protects other antioxidant systems, and participates in cellular regeneration mechanisms [1]. The ascorbic acid molecule contains an enediol group, which underlies its involvement in complex redox reactions and the formation of intermediate products, such as dehydroascorbic acid. However, the effect of ascorbic acid is concentration-dependent. In the presence of free transition metal ions and at high pharmacological concentrations, ascorbic acid may act as a prooxidant exhibiting cytotoxicity [2].

ROS (peroxide, superoxide, and hydroxyl radicals, hydrogen peroxide, and others) are known to be prooxidants, and activate free-radical processes. However, ROS are also capable of recombination (terminate radical reaction chains), and function as signaling molecules involved in cell division, apoptosis, and other processes [3]. ROS concentration increases during particle internalization by immune cells. In a phagocytic cell there are the following processes: the uncoupling of respiratory chains, the depolarization of the inner mitochondrial membrane, and the alterations in the NADH/NAD+ and FAD+ balance [4]. These processes are also the key events necessary for the initiation of chromatin decondensation and the extracellular DNA release [5].

Thus, the internalization activity and extracellular DNA network formation are regulated by NADPH oxidase and redox processes. Therefore, the agents enhancing or inhibiting oxidation–reduction reactions — prooxidants and antioxidants are of biotechnological interest as the regulators of the immune system functioning.

Mononuclear cells — lymphocytes, monocytes, macrophages, natural killer cells, and dendritic cells — constitute a heterogeneous group of leukocytes of the immune system, and are characterized by a single non-segmented nucleus. These cells perform diverse specialized functions within the immune system, including the homeostasis maintenance, the resistance to microorganisms, the participation in inflammatory responses, tissue regeneration, and others [6]. The use of mixed cell suspensions containing different populations of different mononuclear and other cells in *in vitro* screening studies more accurately reflects the complexity of the *in vivo* microenvironment than the use of single cell lines or purified cell subpopulations.

The principal functions of macrophages and monocytes are phagocytosis (a form of endocytosis), antigen processing, and the presentation of antigenic determinants to helper and cytotoxic T lymphocytes. Helper T cells play a central role in transmitting the information about antigen structure to B lymphocytes, while B lymphocytes are transformed into the cells producing the antibodies to the antigen cells. Natural killer cells recognize and eliminate the cells that have lost the ability to express major histocompatibility complex class I molecules [6].

When a high concentration of pathogens, large or small particles of cellular debris is present in the internal environment, immune cells (primarily neutrophils) may activate the formation of extracellular DNA traps as an alternative defense mechanism. DNA networks, or DNA traps, are reticular structures of decondensed chromatin containing histones and antimicrobial enzymes capable of binding to cellular debris and pathogens, and eliminate them [5]. Chromatin decondensation and extracellular DNA network formation are triggered by a cell when endocytosis and phagocytosis become impossible or insufficiently effective.

Particle internalization and extracellular DNA network formation represent two interconnected mechanisms of the innate immunity; they provide effective host protection. Both processes are activated by similar signaling pathways complementing each other, and provide the multilevel protection against pathogens. One of the causes of impaired immune system functioning can be the excessive extracellular DNA network formation by immune cells, which is frequently associated with various pathological conditions as well as chemical exposure and a physical action.

Traditionally, extracellular DNA network formation has been described as a defense mechanism against pathogens, exclusive to neutrophils, and referred to as NETosis [5]. Recent studies have demonstrated that other immune cell types including monocytes, macrophages, eosinophils, mast cells, and lymphocytes, are also capable of generating extracellular DNA networks in response to various stimuli [7–9].

In the context of the present study, it is particularly important to emphasize that the information on extracellular DNA network formation by T and B lymphocytes is extremely limited and the regulation of DNA network formation is largely unexplored. However, these cells are the major components of the mononuclear fractions of blood and spleen, and play the key role in both innate and adaptive immune responses.

The excessive or unregulated formation of extracellular DNA networks by leukocytes contributes to chronic inflammation, tissue damage (including thrombosis and the activation of free radical processes) and, in certain cases, tumor progression [7, 8].

Redox balance is essential for the physiological functioning of immune cells and the maintenance of organismal homeostasis [10]. The formation of extracellular DNA networks and endocytosis are the physiological responses, which are finely regulated by the redox status of the cellular microenvironment [11].

To date, the concentrations of prooxidants and antioxidants (exogenous or endogenous) that modulate the cellular decision between particle internalization and extracellular DNA trap formation remain unexplored. Furthermore, it is of considerable importance to identify the factors and conditions that enable targeted regulation of the mononuclear cell activity.

**The aim of the study** was to identify the regulatory potential of ascorbic acid and HOO• radicals in the formation processes of extracellular DNA networks and the internalization activity of mononuclear cells from rat blood and spleen.

## Materials and Methods

The present *in vitro* study was carried out on mononuclear cells isolated from the blood and spleen of 6 outbred laboratory rats weighing 200–250 g, aged 6–10 months. The animal care conditions were in accordance with standard vivarium requirements.

All experimental procedures were performed in accordance with ethical norms of the Order of the Ministry of Healthcare and Social Development of the Russian Federation “On approval of laboratory practice rules” No.708-n dated August 23, 2010, and the European Convention for the Protection of Vertebrate Animals used for Experimental and Other Scientific Purposes (Strasburg, 1986). The experimental studies were discussed and approved by the Bioethics Committee of National Research Lobachevsky State University of Nizhny Novgorod (protocol No.95 dated February 28, 2025).

**Figure 1.**
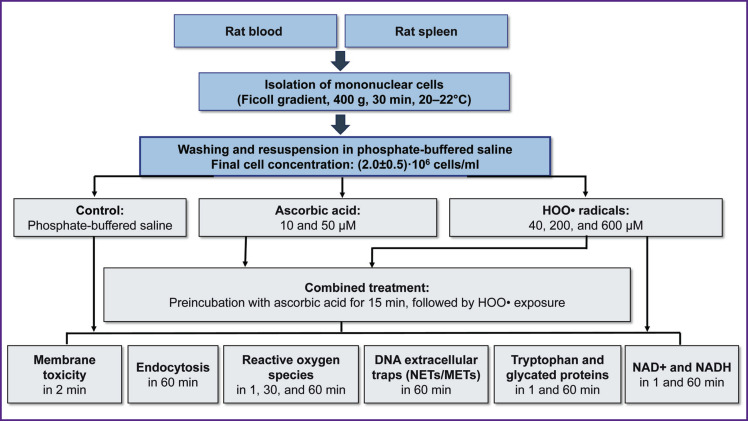
Experimental design

Before collecting the biological material, 100 μl of heparin was administered intraperitoneally to the rats as an anticoagulant, after that the animals were anesthetized with Zoletil 100 (50 mg/kg). The experimental design is illustrated in Figure 1.

### Isolation of mononuclear cells from blood

The blood (8–10 ml) was collected in tubes containing heparin as an anticoagulant. The mononuclear cells were isolated using a density gradient centrifugation with Ficoll. The blood was diluted (1:1) with sterile phosphate-buffered saline (pH 7.4) without Ca^2+^/Mg^2+^ ions at room temperature. Ficoll, 4 ml (1.077 g/ml; Ficoll-Paque®, Russia), was added to a 10-ml tube followed by 8 ml of diluted blood (1:2), which was carefully layered to maintain a clear interface between the two phases (not mixed). The tubes were centrifuged at 3000 rpm (1750 g) for 30 min at room temperature. After centrifugation, four distinct layers were formed: 1) the top layer — plasma; 2) the middle layer — a whitish layer (a ring of mononuclear cells); 3) the bottom layer — Ficoll; and 4) the pellet — red blood cells and granulocytes.

The middle layer of mononuclear cells was carefully collected and transferred to a tube. The collected cells were washed twice with cold (4°C) sterile phosphate-buffered saline at 1500 rpm (875 g) for 10 min, thoroughly removing the supernatant after each centrifugation. To remove residual erythrocytes, the pellet was resuspended in 2 ml of red blood cell lysis buffer (150 mM NH_4_Cl, 10 mM KHCO_3_, 0.1 mM Na_2_EDTA, pH 7.4). Then, 10 ml of phosphate-buffered saline was added, the mixture was centrifuged at 1500 RPM (875 g) for 5 min, and the supernatant was removed. The mononuclear cell pellet was resuspended in phosphate-buffered saline, and the concentration was adjusted to 2.0±0.5·10^6^ cells/ml.

### Isolation of mononuclear cells from spleen homogenate

The spleen was removed under sterile conditions and mechanically homogenized. The suspension was washed with phosphate-buffered saline (pH 7.4) to remove large tissue fragments. Mononuclear cells were isolated according to the protocol described above for blood: centrifugation in a Ficoll gradient (1750 g, 30 min, 20–22°C) followed by double washing with phosphate-buffered saline, the concentration being adjusted to 2.0±0.5·10^6^ cells/ml. The isolation of splenic mononuclear cells in the Ficoll gradient allows the removal of neutrophils, which constitute approximately 15% of the splenic tissue.

The fraction of mononuclear cells is inherently a heterogeneous population including T lymphocytes (CD4^+^/CD8^+^, etc.), B lymphocytes (CD45R^+^), natural killer cells (NK; CD161a^+^) and monocytes (CD68^+^/CD14^+^). In general, in the mononuclear fraction, lymphocytes make up from 70 to 90% of the total number of cells, monocytes — from 10 to 20%, NK cells — from 5 to 15%, and dendritic cells are few, from 1 to 2%.

The heterogeneous fraction was chosen as the initial and necessary step to characterize the overall response of a heterogeneous population to the effects of free radicals, ascorbic acid and their combined effects. It should be recognized that the inherent heterogeneity of the mononuclear fraction eliminates the possibility of studying each cell subpopulation in isolation at this initial stage. However, the analysis of the total effects of suspensions containing various immune cells more accurately models the *in vivo* microenvironment, in which immune cells reside, in contrast to homogeneous subpopulations.

Immediately after the mononuclear cells were isolated, they were exposed to ascorbic acid and HOO• radicals in various concentrations.

Ascorbic acid (Sigma-Aldrich, USA) was used in concentrations of 10 and 50 μM. An equivalent volume of phosphate-buffered saline was added to the control series of cells.

Hydroperoxyl radicals were obtained during the generation of spark discharge plasma radiation using the experimental equipment IR-50 (Lomonosov Moscow State University, Russia). A pulsed spark discharge in the air was generated with a frequency of ~50 Hz; the plasma radiation of the spark discharge is a source of free radicals, primarily HOO• [12].

The concentration of HOO• radicals in an aqueous medium was calculated based on the generation rate of radicals by a generator: (8.0±0.1)·10^–5^ mol/(L/min) [13].The operating time of the spark plasma discharge generator for different doses of HOO• radicals in our study was 0.5, 2.5, and 7.5 min. Accordingly, the concentrations of HOO• radicals were 40, 200, and 600 μM.

To assess the combined effect, the cells were incubated with ascorbic acid for 15 min at room temperature before exposure to free radicals.

The experiment was carried out as follows.

### The assessment of the effect of ascorbic acid and HOO• radicals on cell membrane toxicity (viability)

To assess the membrane-toxic effect of various concentrations of ascorbic acid and HOO• radicals, as well as their combinations, we analyzed the integrity of the plasma membrane (viability). The cells were examined after incubation with the vital dye trypan blue [14]. The principle of the method is that the dye does not penetrate into viable cells with intact membranes. Non-viable cells have damaged membranes, and let the dye enter the cytoplasm and stain the cells blue.

Immediately after treatment, the cell suspensions were mixed 1:1 with a 0.4% trypan blue solution in sterile phosphate-buffered saline. The mixture was incubated for 2 min at room temperature and then placed in a cell counting chamber (Goryaev chamber) for microscopic observation at 40× magnification using a Biomed-4 microscope (BIOMED, Russia). One hundred cells per sample were counted distinguishing between stained (blue, with damaged membranes) and unstained (light, with intact membranes) cells. The results were expressed as a percentage of cells with damaged membranes relative to the total number of cells counted.

Isolated mononuclear cells from 6 animals were used; each experimental series was performed in triplicate (a total of 1800 cells were analyzed).

### Assessment of particle internalization activity (endocytosis)

Endocytosis was used to evaluate the ability of mononuclear cells to internalize latex particles. Monocytes and macrophages internalize particles primarily by phagocytosis; other populations of mononuclear cells (such as T cells and NK cells) and other subpopulations may use alternative mechanisms of endocytosis (such as receptor-mediated endocytosis or macropinocytosis). The heterogeneous mononuclear cell fraction analyzed in this study included mainly T and B lymphocytes, NK cells, monocytes, and macrophages, each with distinct endocytic mechanisms. Thus, the ability to internalize latex particles serves as the most comprehensive indicator of endocytic activity.

The endocytic capacity of mononuclear cells was evaluated *in vitro* by incubation with standardized latex particles.

50 μl of latex particle suspension was added to each tube containing the cell suspension (approximately 2·10^6^ cells). A commercial preparation of latex particles, 1.5 μm in diameter (DIAM, Russia), was diluted 1:100 in sterile phosphate-buffered saline (pH 7.4) to avoid non-specific saturation. The cell-latex mixture was incubated for 60 min at 37°C in a 5% CO_2_ atmosphere, allowing sedimentation and cell-particle contact for active internalization.

After incubation, the cells were carefully centrifuged at 1500 rpm (875 g) for 5 min to remove any non-internalized particles remaining in the supernatant. Thin cell smears were prepared on clean, grease-free slides and allowed to air dry at room temperature. The preparations were then fixed by immersing the slides in 96% absolute ethanol for 20 min.

Differential staining was performed using the Romanovsky method in accordance with the manufacturer’s instructions. This staining technique enables to clearly differentiate the morphology of cells (nucleus, cytoplasm) from the contour of latex particles.

The analysis was performed using an Olympus U-RFL-T optical microscope (Olympus, Japan) with a 100× objective. One hundred mononuclear cells per sample were examined distributed over at least five different microscopic fields of view, to ensure representativeness.

Endocytosis-active cells were considered to be the cells containing one or more intracellular latex particles (identified by their spherical morphology).

To characterize the endocytic response, two main parameters were determined:

endocytic activity — the percentage of active cells calculated as the number of cells, which internalized at least one latex particle divided by the total number of counted cells multiplied by 100;
the endocytic index (uptake capacity) is the efficiency of particle capture by active cells. It was calculated by summing the total number of latex particles internalized by cells counted in all endocytosis-active cells, and dividing this value by the total number of endocytosis-active cells.

### Assessment of intracellular ROS concentration

Intracellular ROS formation during endocytosis was monitored in real time using the fluorescent probe dihydrorhodamine 123 (DHR123). This non-fluorescent probe penetrates cells and, upon oxidation of ROS, is converted to rhodamine 123 — a highly fluorescent fluorophore, the intensity of which is proportional to the intracellular ROS concentration. The analysis is crucial for understanding whether ascorbic acid and the HOO• radical modulate the ability for intracellular ROS generation during endocytosis.

The intracellular concentration of ROS in blood mononuclear cells was analyzed for 1 h (recording interval 30 min) on a Cytoflex flow cytometer (BeckmanCoulter, USA). Briefly, 500 μl of the fluorescent dye (dihydrodamine 123, 1 μM/ml) was added to 50 μl of the mononuclear cell suspension, and incubated for 15 min at room temperature, in the dark. Fluorescence was excited using a blue laser (488 nm), the emission was recorded at 525/40 nm. In each series, the cytometer analyzed the fluorescence of 50,000 cells.

### Assessment of extracellular DNA trap formation

We aimed to evaluate the formation of extracellular DNA networks in the heterogeneous fraction of mononuclear cells from rat blood and spleen exposed to HOO• radicals and ascorbic acid, since extracellular DNA networks formation by these cells reduces the functional activity of the entire mononuclear cell population.

The formation of extracellular DNA networks was observed immediately and 60 min after treating the cells with the studied factors and incubation at 37°. Then, the smears were prepared and stained according to Romanovsky–Giemsa method (CJSC “ECOlab”, Russia). Romanovsky–Giemsa staining is a standard method for visualizing nuclear structures and extracellular DNA networks, as it enables to clearly differentiate decondensed chromatin extending beyond the cell membrane from other cellular components due to the metachromatic staining of nucleic acids in violet-blue tints.

### Assessment of oxidative protein modification by fluorescence of glycated proteins and tryptophan

To assess the effect of ascorbic acid and HOO• radical on the molecular integrity of cell proteins, we measured the intrinsic fluorescence of the amino acid tryptophan and glycosylated proteins.

A decrease in tryptophan fluorescence indicates the molecular denaturation of the amino acid, which alters the structure and function of the protein. Quantitative assessment of glycosylated proteins (end products of glycation, AGEs) enables to determine the intensity of non-enzymatic reactions of glycation and oxidative modification of proteins.

The fluorescence of the amino acid tryptophan was recorded 1 and 60 min after the start of endocytosis using a Fluorat-02-Panorama fluorometer (LLC “Lumex”, Russia) at 288 nm excitation, 350 nm emission; and the fluorescence of glycated proteins at 380 nm excitation, 490 nm emission.

### Assessment of cellular redox balance (NAD+ and NADH levels)

To assess the redox balance of cells, we studied the absorption spectra of the coenzyme NAD+ (oxidized form) and NADH (reduced form) 1 and 60 min after the start of endocytosis using a Fluorat-02-Panorama spectrofluorimeter (LLC “Lumex”, Russia). The absorption spectra were recorded at λ=260 nm for NAD+ and λ=340 nm for NADH. These parameters reflect both the balance of redox processes in a cell and also the metabolic activity.

Due to the scale of the study and the number of required measurements, the experiments were divided between mononuclear cells isolated from blood and spleen. The cell yield obtained from the blood and spleen was sufficient for all planned analyses, but insufficient for simultaneous reproduction of the entire experiment.

In order to optimize the experimental resources, the preliminary comparative analyses were carried out to determine the response of both cell populations according to the parameters of interest. The results of these preliminary studies showed no significant differences between the blood and splenic cells in the assessed parameters.

The membrane-toxic effect of ascorbic acid and HOO• radicals, endocytic activity (particle internalization) and the formation of extracellular DNA networks were studied on the suspensions of splenic mononuclear cells. Endocytic activity (particle internalization), the generation of intracellular ROS, the formation of extracellular DNA networks, the oxidative modification of proteins (the level of glycated proteins and tryptophan), the redox balance of cells (levels of NAD+ and NADH) were studied on the suspensions of mononuclear blood cells.

#### Statistical data processing

We conducted *in vitro* experiments on mononuclear cells from six animals, with each experiment performed in triplicate.

Data processing and analysis were performed using SPSS 25 Statistics software (IBM, USA). Initially, the Shapiro–Wilk test was used to assess the normality of the data distribution.

A one-way parametric analysis of variance (ANOVA) was applied to analyze the intracellular ROS concentration in the mononuclear cells of peripheral blood determined by flow cytometry. The method was chosen due to the large number of events analyzed by the flow cytometer (50,000 cells per sample). The results are presented as M±SD, where M is the arithmetic mean, and SD is the standard deviation of the mean. Tukey’s test was used for post hoc multiple comparisons.

For the experiments where the data did not follow a normal distribution, non-parametric methods were used. The comparisons between the groups were performed using the Kruskal–Wallis test, where p<0.05 was considered statistically significant. The significance levels were adjusted using the Bonferroni correction.

Non-parametric data are presented as box plots showing the median (central line), interquartile range (IQR), as well as minimum and maximum values.

## Results

### The evaluation of the impact of ascorbic acid and HOO• radicals on the membrane toxicity (viability) of cells

The results of the study on the membrane toxic effects of various concentrations of ascorbic acid and the HOO• radical, as well as their combined action, revealed a concentration-dependent damage to the membranes of mononuclear cells caused by both HOO• radicals and high concentrations of ascorbic acid.

**Figure 2.**
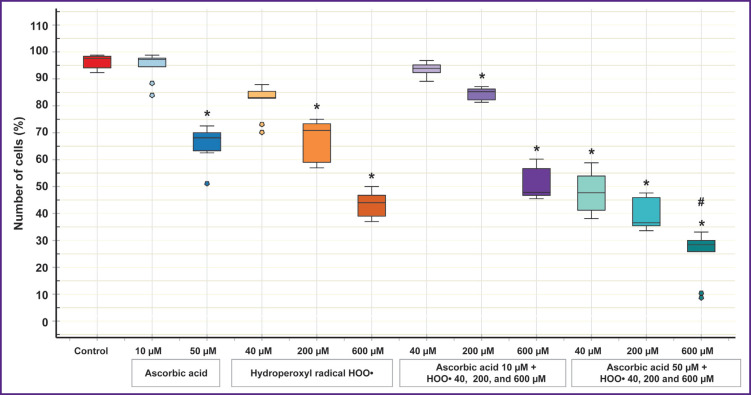
Number of mononuclear cells with intact membranes after incubation with ascorbic acid, exposure to HOO• radicals, and combined exposure to HOO• radicals and ascorbic acid * statistically significant differences compared to the control series (p<0.05); # statistically significant differences compared to the series after exposure to HOO• radicals at a concentration of 600 μM (p<0.05)

High concentrations of HOO• radicals caused membrane damage in 56% of mononuclear cells, while the high concentration of ascorbic acid affected 30% (Figure 2).

The pre-incubation of mononuclear cells with ascorbic acid at a concentration of 50 μM followed by exposure to HOO• radicals demonstrated an increased membrane toxicity effect. Ascorbic acid at this concentration enhanced the destructive action of the radicals on the membranes of mononuclear cells resulting in a pro-oxidant effect.

After determining the membrane toxicity profile at various concentrations of HOO• radicals and ascorbic acid, we assessed the functional capabilities of the mononuclear cells.

### The study of particle internalization activity (endocytosis) by the cells

Endocytosis is a key component of innate immunity and is modulated by various physical and chemical factors, which have an impact on cellular signaling and redox balance in tissues both under normal conditions and during inflammation. Therefore, there were analyzed the influence of ascorbic acid and HOO• radicals on the activity of endocytic reactions and the preservation of functional capabilities by the mononuclear blood cells.

**Figure 3.**
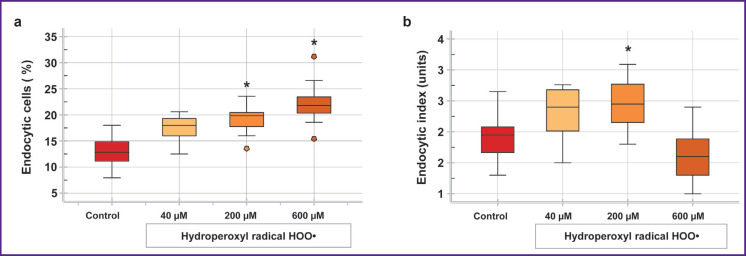
Endocytic (internalization) activity of blood mononuclear cells after exposure to HOO• radicals: (a) number of actively endocytosing cells; (b) endocytic index (an average number of particles internalized per active cell); statistically significant differences compared to the control series (p<0.05)

In the control series, the percentage of active endocytosing cells was 12.84% (IQR=3.7), with the endocytic index varying from 1.3 to 2.65 particles per active cell (Figure 3).

As the concentration of radicals increased, the number of actively endocytosing cells also grew. A concentration of 200 μM increased the endocytic activity by 1.54 times compared to the control series, with uptake capacity reaching up to 3.09 particles per cell. A high concentration of HOO• (600 μM) increased the number of active cells by 1.72 times compared to control values. However, the endocytic index in this series did not differ from the control varying from 1.0 to 2.4 latex particles per cell.

**Figure 4.**
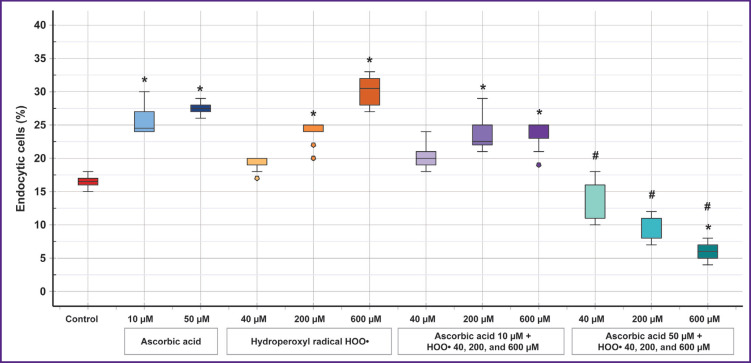
Endocytic (internalization) activity of splenic mononuclear cells after incubation with ascorbic acid, exposure to HOO• radicals, and combined exposure to HOO• radicals and ascorbic acid * statistically significant differences compared to the control series (p<0.05); # statistically significant differences compared to the corresponding doses after treatment with HOO• radicals alone (p<0.05)

The analysis of HOO• effects on endocytic activity of splenic mononuclear cells also revealed a concentration-dependent stimulation (Figure 4).

The exposure to HOO• radicals at a concentration of 600 μM increased the number of endocytosing cells by 1.85 times compared to control, it was 13% higher than the series with mononuclear blood cells.

The incubation only with ascorbic acid (10 μM) (see Figure 4) increased the endocytic activity by 2.05 times compared to the control series.

The combined effect of the radicals and ascorbic acid at a concentration of 10 μM stimulated the endocytic activity of splenic mononuclear cells compared to control values, but was lower than the activity of the cells after incubation with ascorbic acid alone at this concentration.

The pre-treatment of the cells only with ascorbic acid at a concentration of 50 μM activated the endocytic activity; however, in combined exposure, it significantly suppressed the endocytic activity at all studied concentrations of radicals. The combined effect of ascorbic acid at a concentration of 50 μM and the radicals at a concentration of 600 μM reduced the number of actively endocytosing cells by 2.75 times compared to the control series, and by 7.6 times compared to the series after exposure to the radicals at this concentration.

### The study of the intracellular ROS concentration

**Figure 5.**
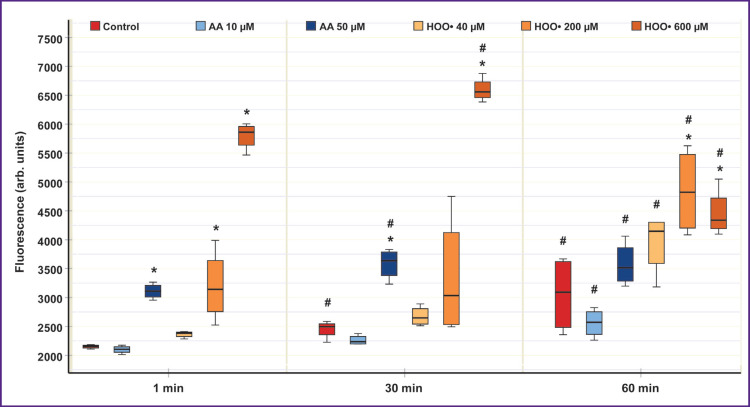
Kinetics of intracellular ROS generation (fluorescence of the intracellular probe dihydrorhodamine 123) by mononuclear cells during 60 min of endocytosis after exposure to ascorbic acid and HOO• radicals AA — ascorbic acid; * statistically significant differences compared to the control series (p<0.05); # statistically significant differences compared to the series after exposure to ascorbic acid and HOO• radicals at 1 min (p<0.05)

We investigated the effect of ascorbic acid and HOO• radicals on intracellular ROS formation during endocytosis in real time. The mononuclear cells in the control series were found to exhibit the characteristic kinetic pattern of a physiological “oxidative burst” during endocytosis. After 60 min from the onset of endocytosis, fluorescence increased by 29.6% (Figure 5). The increase in the probe fluorescence indicated a rise in intracellular ROS concentration during endocytosis.

The incubation of cells with 50 μM ascorbic acid resulted in the significant increase in fluorescence and, consequently, ROS levels at the beginning of endocytosis, 1.5 times higher compared to the control. However, in 60 min, the fluorescence values were similar to those of the control series at the same time point.

The radicals at a concentration of 200 μM caused a sustained hyperactive response with an already elevated baseline fluorescence level. In 60 min, fluorescence increased 1.6 times above control values.

In the cells treated with HOO• radicals at a concentration of 600 μM, significantly higher levels of fluorescence were recorded at the start of the endocytosis process. The values were 2.7 times higher than in the control, indicating the activation of active oxygen species generation within the cell. However, within 60 min, there was observed the decrease in fluorescence levels compared to the beginning of the process, indicating a slowdown in ROS production within the cell.

### The analysis of extracellular DNA network formation

The ability of mononuclear cells to form extracellular DNA networks in response to the studied stimuli was investigated.

**Figure 6.**
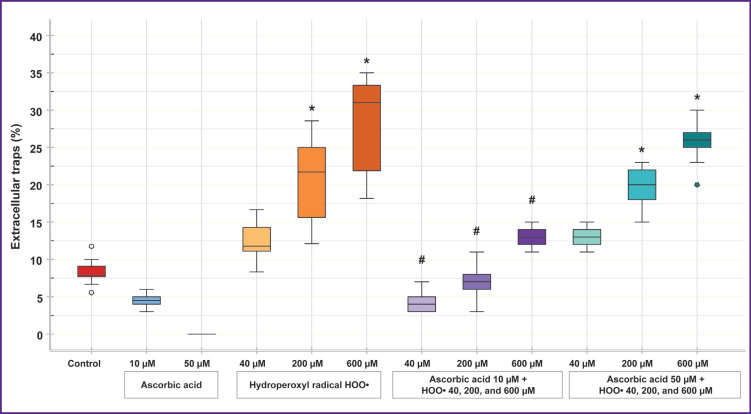
Formation of extracellular DNA traps by mononuclear cells 60 min after incubation with ascorbic acid, exposure to HOO• radicals, and combined exposure to HOO• radicals and ascorbic acid * statistically significant differences compared to the control series (p<0.05); # statistically significant differences compared to the corresponding doses after treatment with HOO• radicals alone (p<0.05)

In the control series, the percentage of blood mononuclear cells forming DNA networks was low (2%, IQR=3). The concentrations of ascorbic acid of 10 and 50 μM did not stimulate the formation of DNA networks (Figure 6). After the exposure to HOO• radicals, a concentration-dependent increase in the number of DNA networks formed by blood mononuclear cells was observed. A concentration of 600 μM HOO• caused a marked increase in the proportion of cells forming DNA networks (19%, IQR=12).

The analysis of extracellular DNA network formation in splenic mononuclear cells showed that in 60 min of incubation, only 7.89% (IQR=2) of cells in the control series formed extracellular DNA networks.

The exposure to HOO• radicals increased the formation of extracellular DNA networks in a dose-dependent manner. HOO• at concentrations of 200 and 600 μM increased the formation of extracellular DNA networks by 2.75 and 3.93 times, respectively, compared to the control series.

When analyzing the combined effect of ascorbic acid and HOO• radicals, we found that the pre-treatment with 10 μM ascorbic acid reduced the formation of extracellular DNA networks induced by HOO• radicals at 200 and 600 μM in a dose-dependent manner by 2.4 and 1.65 times, respectively.

A concentration of 50 μM ascorbic acid completely suppressed the formation of extracellular DNA networks demonstrating an inhibitory effect on chromatin decondensation and DNA network formation. However, the incubation of cells with 50 μM ascorbic acid followed by the exposure to HOO• radicals inhibit no formation of extracellular DNA networks.

### Determining the oxidative modification of cell proteins by the fluorescence level of glycated proteins and tryptophan

To assess the impact of ascorbic acid and the HOO• radical on the structural and functional integrity of membrane and cellular proteins during endocytosis, we measured the intrinsic fluorescence of the amino acid tryptophan. Tryptophan is the major contributor to intrinsic protein fluorescence; a decrease in fluorescence indicates the loosening (denaturation) of the molecule, along with the changes in the structure and function of the protein.

**Figure 7.**
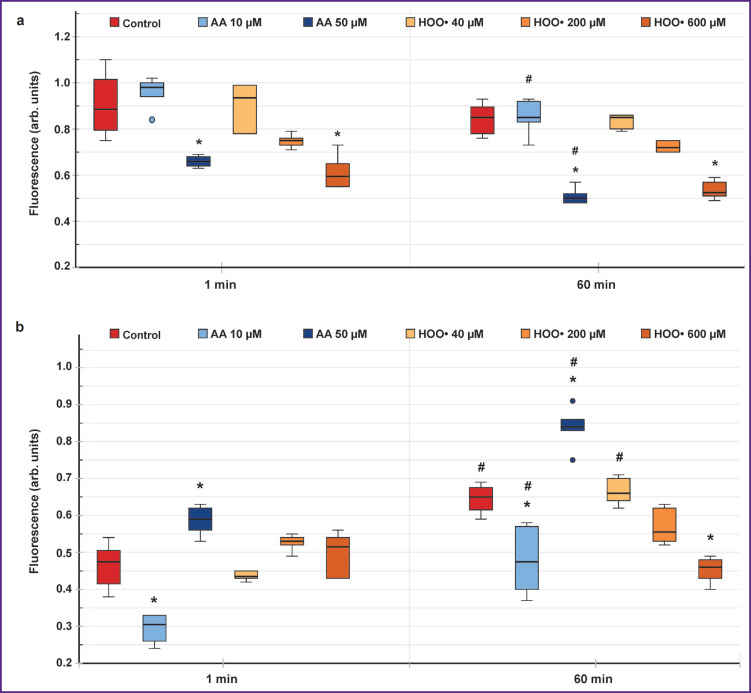
Fluorescence of tryptophan (a) and glycated proteins (b) in mononuclear cells in 1 and 60 min after exposure to ascorbic acid and hydroperoxyl (HOO•) radicals AA — ascorbic acid; * statistically significant differences compared to the control series (p<0.05); # statistically significant differences compared to the corresponding series at 1 min after exposure to ascorbic acid and HOO• radicals (p<0.05)

In the control cells and in the cells treated with 10 μM ascorbic acid, the tryptophan concentration had no significant changes during endocytosis (Figure 7 (a)). In mononuclear blood cells incubated with 50 μM ascorbic acid, a decrease in tryptophan fluorescence of 1.34 times was recorded at the start of endocytosis, and 1.77 times in 60 min compared to the control series.

After exposure to the highest concentration of HOO• radicals (600 μM), tryptophan fluorescence decreased by 1.59 times at the beginning of endocytosis (p<0.01) and by 1.62 times in 60 min (p<0.01) compared to the control.

To evaluate the extent of molecular modification of the membrane proteins in mononuclear cells, we quantitatively determined the specific fluorescence related to glycated proteins, which is typically associated with damage to protein complexes and the formation of advanced glycation end products resulting from non-enzymatic reactions between reducing carbohydrates (capable of participating in reduction reactions) and the amino groups of proteins.

In the control group, 60 min after the induction of endocytosis, we observed an increase in fluorescence of glycated proteins by 1.4 times compared to the baseline level (Figure 7 (b)).

In the cells incubated with 10 μM ascorbic acid, the fluorescence of glycated proteins decreased by 1.4 times compared to the control at both 1 and 60 min after the induction of endocytosis. In contrast, in the cells treated with 50 μM ascorbic acid, an increase in fluorescence of 1.3 times compared to the control was observed at both time points (1 and 60 min).

The exposure to HOO• radicals had no significant impact on the fluorescence of glycated proteins 1 min after the start of endocytosis. However, in 60 min, a concentration of HOO• 600 μM reduced the fluorescence by 1.4 times compared to the control group. This may be due to the inhibition of glycation reactions, radical degradation of the resulting products, or the disruption of intermediate protein metabolism.

### The analysis of the redox balance in cells (NAD+ and NADH levels)

To assess the impact of ascorbic acid and the HOO• radical on cellular metabolism, we examined the absorption spectra of the coenzymes NAD+ and NADH. These parameters reflect the balance of oxidative and reductive processes in a cell.

**Figure 8.**
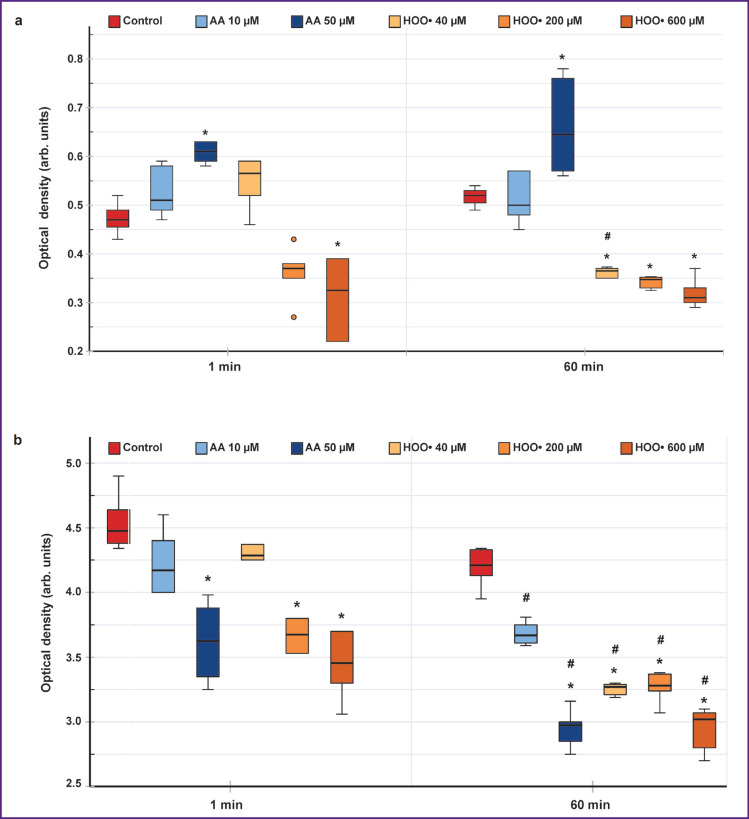
NAD+ (a) and NADH (b) levels in mononuclear cells in 1 and 60 min after exposure to ascorbic acid and HOO• radicals AA — ascorbic acid; * statistically significant differences compared to the control series (p<0.05); # statistically significant differences compared to the corresponding series at 1 min after exposure to ascorbic acid and hydroperoxyl (HOO•) radicals (p<0.05)

The analysis of NAD+ level revealed treatment-dependent changes (Figure 8 (a)). In the control group, NAD+ levels remained stable.

The cells treated with 50 μM ascorbic acid showed the greatest increase in NAD+ levels, by 1.30 times in 1 min and by 1.24 times in 60 min compared to the control series.

In contrast, the exposure to HOO• radicals caused the significant decrease in NAD+ concentration, i.e., the oxidized form of the coenzyme. In 60 min, NAD+ levels decreased by 1.45, 1.54, and 1.67 times at 40, 200, and 600 μM HOO•, respectively, compared to the control.

The assessment of NADH concentration indicated that in the cells treated with 50 μM ascorbic acid, there was a decrease in NADH concentration by 1.22 times at the onset of endocytosis and by 1.41 times in 60 min compared to the control series (Figure 8 (b)).

The exposure to HOO• radicals also caused a decrease in NADH concentration, i.e., the reduced form of the coenzyme. In the series with a radical concentration of 600 μM, a reduction of 1.39 times was observed in 60 min compared to the control series.

These results indicate that decreased NADH levels reflect a shift in redox balance toward oxidation, particularly after incubation with 50 μM ascorbic acid.

## Discussion

The analysis of the regulatory potential of HOO• radicals, ascorbic acid, and their combination is the initial step for studying the regulation processes involved in the formation of DNA networks and the internalization activity of a heterogeneous population of mononuclear cells. The use of suspensions of multiple cells more accurately reflects the complexity of the immune microenvironment *in vivo* than the use of homogeneous cell lines or purified subpopulations.

### The assessment of the effects of ascorbic acid and HOO• radicals on membrane toxicity (viability) of cells

In the present study, the integrity of the plasma membrane evaluated by trypan blue staining indicated that the mononuclear cells exposed to ascorbic acid and HOO• radicals cause dose-dependent membrane toxic effects.

At low concentrations (10 μM), ascorbic acid had no effect on membrane integrity acting as a water-soluble antioxidant. Being hydrophilic, ascorbic acid does not penetrate the lipid bilayer, and its effect on lipids is indirect [15, 16].

At low concentrations, ascorbic acid participates in capture reactions of single radicals formed under normal conditions and is absorbed by the cell before it can interact with transition metal ions [16]. In contrast, at high concentrations (50 μM), ascorbic acid reduces Fe^3+^ to Fe^2+^, subsequently activating the Fenton reaction (Fe^2+^ + H_2_O_2_ → Fe^3+^ + HO• + OH^–^). The resulting HO• radicals (E°' ≈ +2.31 V) interact with the hydrogens of polyunsaturated fatty acids initiating a chain reaction of lipid peroxidation that disrupts membrane fluidity, creating hydrophilic pores or ruptures approximately 2.0–2.5 nm in size, which is the effective diameter for trypan blue penetration into the cell [2, 17, 18].

Our findings are consistent with the literature data that the cytotoxicity of high concentrations of vitamin C is due to excessive H_2_O_2_ formation within the cell through the catalytic reduction of iron ions [19].

HOO• radicals represent the protonated form of the superoxide anion. Unlike ascorbate, HOO• is a neutral and moderately lipophilic compound [20].

HOO• exhibited the membrane toxicity at concentrations ≥200 μM. The nature of the HOO• radical allows it to diffuse through the bilayer and directly interact with hydrogen from polyunsaturated fatty acids, initiating peroxidation processes. HOO• radicals trigger chain reactions of lipid and protein oxidation, binding and removing hydrogen atoms from polyunsaturated fatty acids in the membranes, creating self-sustaining cascades of free radical damage [21, 20]. Furthermore, HOO• can directly oxidize thiol residues of transmembrane proteins altering their conformation and disrupting their function. For example, in mitochondria, radical reactions damage cardiolipin and initiate cell death through apoptosis [22, 23].

In combination, low concentrations of ascorbic acid protect membranes from the action of radicals. At the water-lipid interface, ascorbic acid in its monoanionic form (ascorbate, AH^–^) donates a hydrogen atom to peroxyl radicals (LOO•), it resulting in the formation of a semi-dehydroascorbate radical (A^–^•), which is stabilized by the resonance between the oxygen and the conjugated carbonyl double bond, thus protecting membrane lipids from exogenous HOO• and endogenous peroxide oxidation. Ascorbic acid acts as an antioxidant. Moreover, ascorbic acid stimulates the glutathione system through dehydroascorbate reductase contributing to the maintenance of the cellular redox status [16, 24, 25].

The combination of ascorbic acid and HOO• at high concentrations enhanced the membrane-toxic effect, which can be explained by the intensification of the iron redox cycle, in which HOO• mobilized Fe^3+^ from ferritin, while ascorbic acid reduces it to Fe^2+^, generating numerous highly reactive chemical species HO• [26].

The gradual decrease in the number of viable cells indicates that as the concentration of ascorbic acid and HOO• increases, the oxidative imbalance begins to overwhelm the cellular defense mechanisms. In this situation, the antioxidant system can no longer neutralize free radicals, leading to the activation of free radical processes and the formation of more pores in the membrane [27].

### The study of particle internalization activity (endocytosis) by cells

Despite the membrane-toxic effect, ascorbic acid and HOO• radicals independently stimulated the processes of particle internalization by cells (endocytosis).

Ascorbic acid protects thiol groups in proteins (clathrin Cys 43, dynamin Cys 607, Rab5 Cys 81) involved in endocytosis, while simultaneously generating sublethal concentrations of H_2_O_2_, which activate AMPK and ERK1/2 pathways, phosphorylating Rab5 and rabinosin-5, as well as the regulatory mechanisms of endocytosis and endosome fusion [28, 29].

Despite its membrane-toxic effect HOO• activates compensatory processes, which stimulate particle internalization. HOO• radicals reversibly oxidize phosphatases (PTENCys 124, PTP1BCys 215) to sulfenic acid (–SOH) resulting in their inactivation and ensuring sustained signaling to the nucleus (PI3K/Akt). Additionally, HOO• radicals activate actin polymerization processes (Rac1/Cdc42) [30, 31].

The combined action of high concentrations of ascorbic acid and HOO• radicals inhibited endocytosis. It is likely to be related to the irreversible oxidation of endocytic cysteines to sulfinic (–SO_2_H)/sulfonic (–SO_3_H) acid by HO• groups formed in ascorbic acid excess, or as a result of reactions with HOO• radicals. Moreover, lipid peroxidation processes activated under these conditions increase the membrane stiffness, hindering invagination processes and reducing the internalization activity of cells [32, 33].

The comparison of endocytic activity of mononuclear cells in peripheral blood and spleen revealed the quantitative differences in their activity under the studied conditions. The splenic cells showed more pronounced endocytosis stimulation when exposed to HOO•. This difference demonstrates the functional heterogeneity of macrophage populations: the splenic macrophages specialize in capturing not only pathogens, but also damaged particles and cells of their own organism, and can have the more powerful phagocyte mechanism or more effective antioxidant systems [34, 35].

### The study of intracellular ROS concentration

The real-time monitoring of intracellular ROS production revealed the patterns directly correlated with mononuclear cell function — a dose-dependent increase in ROS. This is the classic profile of an oxidative burst: a gradual and sustained increase in ROS within 60 min after stimulation reflects coordinated activation of NADPH oxidase and other ROS-generating systems required for particle internalization [36].

In the cells incubated with high concentrations of ascorbic acid, the mechanism for increasing ROS concentration is indirect and depends on the presence of metals variable valency: HO• generated by Fenton reaction in extracellular space, oxidizes membrane lipids to form LOO• radicals, which diffuse into a cell, and also generates hydrogen peroxide, which penetrates through the aquaporins [37].

On the other hand, the mechanism of ROS increase using HOO• is direct: due to its lipophilicity HOO• diffuses into the cytosol; participates in such reactions as oxidizing NADH to NAD+ to form H_2_O_2_; mobilizes Fe^2+^ from ferritin and Fe-S centers catalyzing intracellular Fenton reactions; damages mitochondria causing the leakage of mitochondrial ROS [38].

It is important to note the change in the kinetics of intracellular free radical formation observed in the cells treated with high HOO• concentrations: high initial levels of ROS (2.7 times higher than in the control group) were followed by a decrease in 60 min indicating a decrease in the activity of cellular oxidases. This functional depletion coincides with the maximal induction of extracellular DNA network formation, membrane damage, and cytotoxicity [39]. A decrease in intracellular ROS levels at the final stages of endocytosis may reflect the depletion of metabolic substrates, the oxidative inactivation of NADPH oxidase due to protein damage, or a transition to cell death programs, which consume cellular resources and disrupt metabolism [40]. It is noteworthy that the same kinetic profile (a high baseline level and a subsequent decrease in ROS concentration) was described in neutrophils undergoing NETosis, where sustained ROS formation is necessary for chromatin decondensation [41].

Ascorbic acid did not activate the formation of extracellular DNA networks at any of the concentrations studied. Mohammed et al. [42] showed ascorbic acid to reduce the expression of mRNA peptidyl arginine deiminase 4 (PAD4) and block the activation of signaling pathways necessary for the formation of extracellular DNA networks. In this regard, we suggest that ascorbic acid, by suppressing PAD4 expression, can reduce histone citrullination and, consequently, chromatin decondensation. Activated T lymphocytes in the inflammatory microenvironment are known to be able to release mitochondrial DNA acting as a DAMP and enhance inflammation through TLR9 receptors [43, 44].

Clinical studies have found that the intravenous administration of ascorbic acid to patients with acute respiratory distress syndrome caused by sepsis reduces the severity of the disease by reducing the excessive formation of extracellular neutrophil traps [45].

HOO• dose-dependently increases the formation of extracellular DNA networks. The mechanism includes PAD4 activation. HOO• oxidizes regulatory cysteines (Cys 409, 645) and stimulates the influx of Ca2^+^ by activating calcium channels necessary for the work of enzymes involved in DNA release. PAD4 activates citrulline and histones H3/H4 decondensing chromatin [43, 46].

We showed that the exposure to HOO• radicals at a concentration of 600 μM led to the activation of the formation of extracellular DNA networks, which correlate with the present study findings on the damage to cell membranes. This fact enables to consider the hydroperoxyl radical as a physiologically significant inducer of extracellular DNA networks due to its ability to cause free radical damage to cytoplasmic, nuclear, and other membranes [47].

One of the most important findings of our research is the revealed ability of ascorbic acid at low concentrations to significantly inhibit the formation of extracellular DNA networks induced by HOO• radicals. It demonstrates the chemical neutralization of HOO• by ascorbic acid, the maintenance of the reduced (–SH) state of PAD4 cysteines by intracellular ascorbic acid and inhibition of PAD4 [48].

Such a protective effect assumes that ascorbic acid neutralizes the radicals and thus — free radical reactions, which are necessary to activate the intracellular molecular pathways to form extracellular DNA networks [16]. In clinical practice, ascorbic acid is used as an inhibitor of the increased formation of extracellular DNA networks in patients with autoimmune and inflammatory diseases, when the excessive formation of extracellular traps is a biomarker characterizing the disease severity [49].

The combination of high concentrations of ascorbic acid with HOO• radicals resulted in the formation of extracellular DNA networks similar to those produced when exposed to HOO• alone. Enhanced iron redox cycling activated under these conditions cause PAD4 to oxidize irreversibly in cells and the nucleus damage that contributes to DNA release. The findings reflect the concentration-dependent potential of the ascorbic acid action [2].

### Determining the oxidative modification of cell proteins by the fluorescence level of glycated proteins and tryptophan

The oxidative modification of proteins was evaluated using two spectroscopic markers. The fluorescence of tryptophan and glycosylated proteins reveals the molecular signs of protein damage [50]. The tryptophan intrinsic fluorescence is an indicator of protein conformational and functional condition. The fluorescence of tryptophan was found to reduce in the presence of high concentrations of ascorbic acid and HOO• radicals.

High concentrations of ascorbic acid, as mentioned above, activate Fenton reaction by generating a hydroxyl radical. It attacks the indole ring of tryptophan forming indole hydroperoxide, which is decomposed into N-formylkynurenine (NFK), or is carried by the hydrogen atom from the N-H indole bound and other non-luminescent derivatives [51].

The exposure to high concentrations of HOO• radicals caused a rapid decrease in the tryptophan fluorescence indicating the protein damage since the onset of the radical exposure. The effect of HOO• is due to its ability to penetrate through a membrane and reach intracellular proteins, as well as to activate DNA network formation pathways [52].

The glycosylated protein fluorescence increased at high and decreased at low ascorbic acid concentrations and high HOO• concentrations.

Reducing protein glycation reactions suggests that ascorbic acid at low concentrations not only prevents direct damage from free radicals but also inhibits non-enzymatic glycation reactions, which naturally occur during particle internalization [53]. In contrast, at high concentrations, ascorbic acid is oxidized to dehydroascorbic acid, which is much more reactive, and reacts with lysines forming fluorescent advanced glycation end-products (AGEs). The findings indicate that the predominant damage mechanism is glyco-oxidation, i.e., the formation of pyrrole-lipid-amino acid complexes involving arginine, lysine, and pentoses/riboses [54].

The increase in glycation products leads to the activation of NADPH oxidase generating superoxide anion radicals, and the formation of lipid and membrane phospholipid peroxides. Such molecular modifications correlate with moderate cytotoxicity, membrane damage, partial formation of extracellular DNA networks, and the inhibition of endocytosis [55].

The exposure to high concentrations of HOO• radicals resulted in decreased glycated protein levels at 60 min. This pattern suggests the degradation of these proteins due to an excess of free radicals or the inhibition of their formation due to the depletion of carbonyl precursors, which are redirected by the cell to other metabolic pathways [56]. This damage profile correlates with high cytotoxicity, cellular membrane damage, intense formation of extracellular DNA networks, and the changes in endocytosis efficiency.

### The analysis of the redox balance in cells (NAD+ and NADH levels)

The concentrations of NAD+ and NADH act as an integrated sensor of the cellular redox and metabolic state, and the imbalance in this sensor can determine the transition between functional programs, such as efficient endocytosis and DNA network formation.

With ascorbic acid, we observe an increase in NAD+ and a decrease in NADH resulting in an increased NAD+/NADH ratio. The increase in NAD+ reflects the direct oxidation of NADH to NAD+ by the A•^–^ radical (semi-dehydroascorbate) and the resulting H_2_O_2_, and/ or activation of the pentose phosphate pathway, which enhances the production of NADPH and NAD+ [57]. The decrease in NADH is explained by mitochondrial damage caused by HO• (peroxidative oxidation of cardiolipin, complex I inactivation), which reduces NADH production in the Krebs cycle and the respiratory chain. Thus, high levels of ascorbic acid create a state of hyperoxidation (high NAD+ levels, low NADH levels) characteristic of impaired mitochondrial metabolism, but with the potential for NAD+ regeneration [58].

At high levels of HOO•, we observed simultaneous decrease in both NAD+ and NADH levels. The findings may reflect massive activation of PARP1, which uses NAD+ for cellular repair processes following the cell damage induced by HOO• [59]. Additionally, HOO• directly oxidizes NADH contributing to its decreased levels. Mitochondrial damage (peroxidative oxidation of cardiolipin, inactivation of aconitase, and complex I inactivation) further reduces NADH production [60]. Finally, NAMPT inactivation through the oxidation of its catalytic cysteine (Cys 263) blocks the synthesis of NAD+ from nicotinamide hindering recovery. The depletion of NAD+ reserves is particularly critical, since this molecule is essential for glycolysis and adenosine triphosphate generation, which explains the inability to sustain energy-demanding functions [61]. The result is a collapse of the overall NAD+ and NADH pool corresponding to energy depletion and cell death in the process of extracellular DNA network formation.

### Study limitations

Despite the analysis of the heterogeneous reactions of the mononuclear cell fraction to the stimuli under study enables to assess the integral response of the population, it is necessary to mention a number of limitations of the present study. The use of a heterogeneous fraction of mononuclear cells does not allow for isolating the contribution of individual subpopulations to the observed effects. In addition, the study was performed *in vitro*, which does not fully reproduce the complexity of the immune microenvironment *in vivo*. The experiments using animal models will also be carried out to extent the understanding of the obtained *in vitro* effects.

## Conclusion

Our findings are significant for understanding the physiology of the immune response. The optimization of antioxidant activity may enhance the functioning of phagocytes in patients with immunodeficiency conditions or generalized infections.

High concentrations of ascorbic acid inhibiting phagocytosis indicate potential adverse effects of elevated levels of ascorbic acid under certain conditions, particularly when combined with active free radical processes and in certain pathologies (sepsis, radiation disease, neoplastic and neurodegenerative processes, etc.).

The ability to induce a controlled gradient of redox reactions through endogenous free radicals enables to model the threshold values for functional transitions providing a valuable tool for toxicological and immunomodulatory corrections and studies.

The capacity of HOO• radicals to activate the formation of DNA networks by mononuclear cells from the spleen and blood is a finding that may be applied to regulate this process in the treatment of hospital-acquired and other infections, when enhanced pathogen clearance is required. The ability of ascorbic acid to block the formation of DNA networks might also find its application in correcting metastasis processes in certain neoplastic conditions. The findings may be relevant for developing immunomodulatory and regulatory strategies for both immunodeficiency and autoimmune processes.
